# An Interobserver Reproducibility Analysis of Ki67 Visual Assessment in Breast Cancer

**DOI:** 10.1371/journal.pone.0125131

**Published:** 2015-05-01

**Authors:** Ruohong Shui, Baohua Yu, Rui Bi, Fei Yang, Wentao Yang

**Affiliations:** 1 Department of Pathology, Fudan University Shanghai Cancer Center, Fudan University, Shanghai 200032, China; 2 Department of Oncology, Shanghai Medical College, Fudan University, Shanghai 200032, China; University of Toronto, CANADA

## Abstract

**Background:**

Ki67 labeling index (LI) is used as a predictive marker and is associated with prognosis in breast cancer. However, standardised methodologies for measurement are lacking which has limited its application in clinical practice. In this study, we evaluated the interobserver concordance of visual assessment of Ki67 LI in breast cancer.

**Methods:**

Ki67- immunostained slides of 160 cases of primary invasive breast cancer were visual assessed by five breast pathologists with two different methods to choose the scoring fields: (1) hot-spot score, (2) average score. Proportions of positive invasive tumor cells at 10 % intervals were scored. The intra-class correlation coefficient (ICC) was used to assess the interobserver reproducibility.

**Results:**

(1) A perfect concordance of Ki67 LI was demonstrated according to both score methods (*P*<0.0001). Average score method (ICC, 0.904) demonstrated a better correlation than hot-spot score method (ICC, 0.894). (2) By respective means according to two score methods, all cases were classified into three groups (≤10%, 11%-30% and >30% Ki-67 LI). The concordance was relatively low in intermediate Ki67 LI group compared with low and high Ki67 LI groups. (3) All cases were classified into three groups by paired-difference (d) between means of hot-spot score and average score (d<5, 5≤d<10, d≥10). The consistency was observed to decrease with increasing paired-difference according to both methods.

**Conclusions:**

Visual assessment of Ki67 LI at 10 % intervals is a candidate for a standard method in breast cancer clinical practice. Average score and hot-spot score of visual assessment both demonstrated a perfect concordance, and an overall average assessment across the whole section including hot spots may be a better method. Interobserver concordance of intermediate Ki67 LI in which most cutoffs are located for making clinical decisions was relatively low.

## Introduction

Uncontrolled proliferation is an important feature of malignant tumors. The nuclear proliferation marker Ki67 is used as a predictive marker for response to chemotherapy and is associated with patient prognosis in breast cancer [1–7]. Assessment of Ki67 labeling index (LI) by immunohistochemical staining has been widely used in pathological evaluation of breast cancer clinical practice.

In 2011, the 12^th^ St. Gallen Consensus Meeting suggested that the Ki67 LI is important for distinguishing between ‘‘luminal A” and ‘‘luminal B (HER2-negative)” breast cancer subtypes and advised adjuvant chemotherapy for luminal B but not for luminal A [8]. Therefore, the standardization of Ki67 assessment is considered more crucial because of its values in clinical practice. However, Ki67 LI measurements by immunohistochemical analysis lack interobserver and interlaboratory reproducibility [9–13]. Currently, there is still no global guideline, with both reproducibility and objective standardization, has been established for Ki67 LI assessment. Standardised methodologies for measurement and cutoff points for Ki67 are lacking which has limited its evaluation and application in clinical practice.

Manual counting of as many as 1000 cancer cells is often recommended to evaluate Ki-67 LI [14], but this method is labor intensive and error prone. In this study, we evaluated the interobserver concordance of visual assessment of Ki67 LI of breast cancer among five breast pathologists and to discuss the potential value of visual assessment in clinic practice.

## Materials and Methods

### Tissue specimens and immunohistochemical staining

One hundred and sixty cases of primary invasive breast cancer were extracted from the pathology database of Fudan University Shanghai Cancer Center. An important object of this study was to evaluate the interobserver concordance of visual assessment in intermediate Ki67 LI group in which most cutoffs are located for making clinical decisions. Since most of grade 1 breast cancers are ER/PR strongly positive cases with low Ki67 LI, Ki67 LI is not crucial in decision-making of the treatment in these patients. So we included less grade 1, but more grade 2 and grade 3 cases of invasive breast cancer in this study. All patients underwent surgery at the Cancer Center in 2012 without neoadjuvant chemotherapy. Clinicopathological features of all cases were reviewed. All specimens were fixed with 10% neutral phosphate-buffered formalin and paraffin-embedded. 4μm-thick slices of representative tumor blocks were stained with hematoxylin and eosin (H&E) and stained on Benchmark XT system (Ventana, Tucson, AZ, USA). MIB-1 clone antibody was used (1:100; Code M7240, Dako, Glostrup, Denmark) for Ki67 staining.

### Evaluation of Ki67 LI

**Fig 1:**
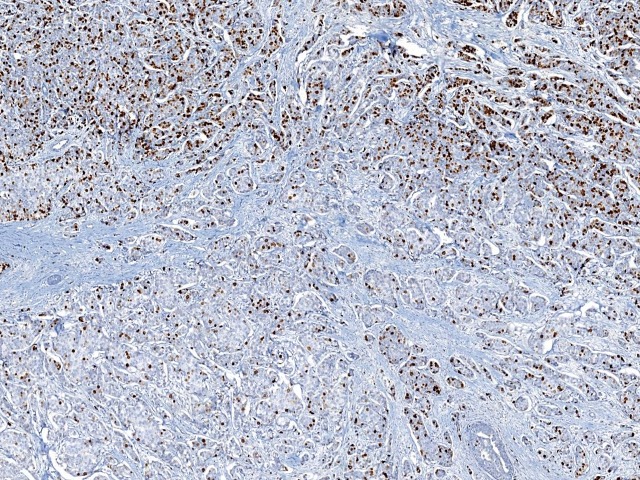
A heterogeneously stained case of Ki67. Ki67 LI was high in upper areas and low in lower areas.

**Fig 2:**
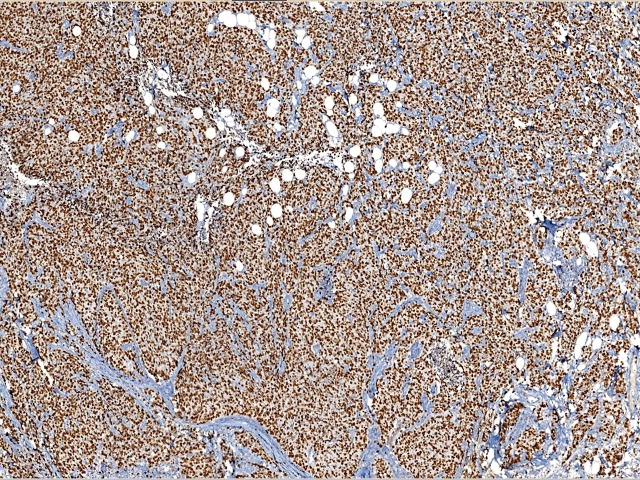
A homogenously stained case of Ki67. High Ki67 LI was diffuse and homogenous across the slide.

Ki67 assessment was performed by five breast pathologists independently, in a blinded manner. Our pathology department has been a highly specialized center and the five observers included in this study were all experienced breast pathologists. Specific instructions were given to the five pathologists for Ki67 assessment. For each case, an H&E slide from the same paraffin block was used for morphology observation. Nuclear staining of any intensity was defined as Ki67 positive. The Ki67 LI was visually scored for the percentage of nuclei positive tumor cells among all tumor cells in invasive tumor area. The whole slide was scanned under low-power microscopy first. At least three high-power (×40 objective) fields should be selected to represent the spectrum of staining seen on initial overview of the whole slide. In heterogeneously stained samples (Fig 1), each pathologist used two different methods to choose the scoring fields: (1) hot-spot score: the pathologists focused on the areas of hot spots, defined as areas in which Ki67 staining is particularly prevalent, and at least three independent areas were selected, hot spots distributed in the invasive edge of the tumor must be included; (2) average score: the pathologists selected at least three independent areas including hot spots in an overall average assessment across the section. If the staining was homogenous (Fig 2), at least three randomly high-power fields were scored. The hot-spot score and average score were same in such samples. All slides were visually estimated, and proportions of positive cells were scored at 10% intervals (i.e., 10, 20, 30, 40, 50, 60, 70, 80, 90, 100%).

### Statistical analysis

To assess the interobserver reproducibility of Ki67 LI according to two score methods, the intra-class correlation coefficient (ICC) was estimated with a 95% confidence interval (CI) using two-way random models. Higher ICC usually indicates better consistency. There is no universally accepted standard criteria for the ICC, the following criteria similar to the kappa coefficient were used in our study [15]: 0.00–0.20 was interpreted as “slight correlation”; 0.21–0.40 as “fair correlation”; 0.41–0.60 as “moderate correlation”; 0.61–0.80 as “substantial correlation”; and >0.80 as “almost perfect correlation.” Two-sided *P* value less than 0.05 was considered to be statistical significant. The computations were done with the statistical software package SPSS 19.0 for Windows (SPSS Inc., Chicago, IL, USA).

### Ethics Statement

Our study was approved by Ethics Institutional Review Board of Fudan University Shanghai Cancer Center. The patient records/information was anonymized and de-identified prior to analysis.

## Results

### Clinicopathological data

Clinicopathological features of 160 cases of primary invasive breast carcinoma were summarized in Table 1. All patients were female and ranged in age from 23 to 93 years, with a median age of 51. The tumors ranged from 0.5 to 5.0 cm in size, with a median diameter of 2.3 cm. 152 cases were diagnosed as invasive carcinoma of no special type, 6 cases were invasive lobular carcinoma, and 2 cases were mucinous carcinoma. Invasive tumors were grade 1 in 6 cases, grade 2 in 83 cases and grade 3 in 71 cases. 122 cases were positive for estrogen receptor (ER), 108 cases were positive for progesterone receptor (PR), and 38 cases were positive for human epidermal growth factor 2 (HER2). According to TNM stage, 46 cases were classified as stage I, 92 cases were stage II, and 22 cases were stage III.

**Table 1:** Clinicopathological features of 160 cases of invasive breast cancer.

	N (%)
**Histologic type**	
invasive carcinoma of no special type	152 (95.0)
invasive lobular carcinoma	6 (3.8)
mucinous carcinoma	2 (1.2)
**Histologic grade**	
1	6 (3.8)
2	83 (51.9)
3	71 (44.3)
**ER**	
+	122 (76.2)
−	38 (23.8)
**PR**	
+	108 (32.5)
−	52 (67.5)
**HER2**	
+	38 (23.8)
−	122 (76.2)
**TNM Stage**	
I	46 (28.7)
II	92 (57.5)
III	22 (13.8)
IV	0 (0)

For ER and PR, nuclear staining in ≥1% of the tumor cells was considered positive.

For HER2, HER2 protein overexpression (3+) by immunohistochemical staining or HER2 gene amplification by FISH detection was considered positive.

### Evaluation of Ki67 LI

The detailed data of scores of each pathologist was provided in S1 Dataset. A perfect correlation of Ki67 LI among five pathologists was both demonstrated according to two score methods (*P*<0.0001) (Table 2). Average score method (ICC, 0.904 {95% CI 0.881, 0.925}) demonstrated a better correlation among five pathologists than hot-spot score method (ICC, 0.894 {95% CI 0.869, 0.916}).

**Table 2:** The ICC and 95% CI on Ki67 LI by average score and hot-spot score.

Score method	ICC(95% CI)	F	*P*
Average score	0.904(0.881, 0.925)	48.291	<0.0001
Hot-spot score	0.894(0.869, 0.916)	43.250	<0.0001

**Fig 3:**
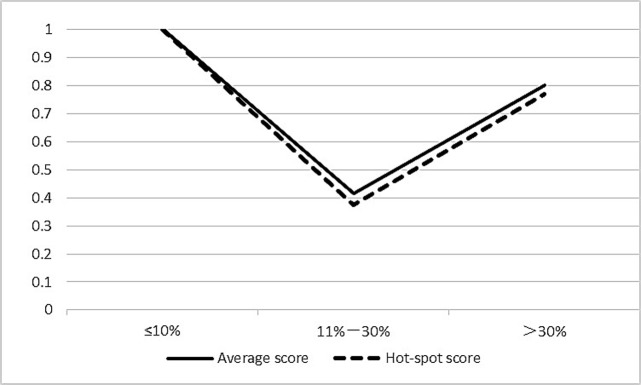
The correlations between ICC and respective KI67 LI means according to two score methods. The ICC was relatively low in intermediate Ki67 LI group (11%-30%) compared with low (≤10%) and high (>30%) Ki67 LI groups according to both methods. X axis: means of Ki67 LI according to two score methods; y axis: ICC.

Most of the proposed cutoff values of Ki67 for making clinical decisions are between 10%-30%. By respective means according to two score methods, all cases were classified into three groups (≤10%, 11%-30% and >30% Ki-67 LI) to allow categorical data analyses. The ICC was relatively low in intermediate Ki67 LI group (11%-30%) compared with low (≤10%) and high (>30%) Ki67 LI groups according to both methods (Table 3; Fig 3). Average score method showed a perfect correlation in ≤10% Ki-67 LI group (ICC, 1.000 {95% CI 1.000, 1.000}), a moderate correlation in 11%–30% Ki-67 LI group (ICC, 0.415 {95% CI 0.300, 0.541}) and a substantial correlation in >30% Ki-67 LI group (ICC, 0.800 {95% CI 0.730, 0.861}). Hot-spot score method showed a perfect correlation in ≤10% Ki-67 LI group (ICC, 1.000 {95% CI 1.000, 1.000}), a fair correlation in 11%–30% Ki-67 LI group (ICC, 0.376 {95% CI 0.244, 0.527}) and a substantial correlation in >30% Ki-67 LI group (ICC, 0.770 {95% CI 0.705, 0.829}). Interobserver reproducibility of 11%-30% Ki67 LI group was both relatively poor according to two score methods.

**Table 3:** The ICC and 95% CI on Ki67 LI, stratified by means of average score and hot-spot score.

Means	Average score		Hot-spot score	
	N	ICC (95% CI)	N	ICC(95% CI)
≤10%	32	1.000 (1.000, 1.000)	25	1.000 (1.000, 1.000)
11%−30%	64	0.415 (0.300, 0.541)	47	0.376 (0.244, 0.527)
>30%	64	0.800 (0.730, 0.861)	88	0.770 (0.705, 0.829)

**Fig 4:**
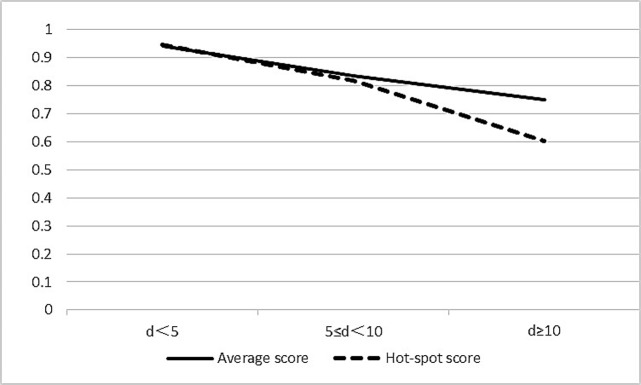
The correlations between ICC and paired-difference between Ki67 LI means of two score methods. The ICC was observed to decrease with increasing paired-difference between means of hot-spot score and average score. X axis: paired-difference (d) between Ki67 LI means of two score methods; y axis: ICC.

To evaluate the correlation between Ki67 staining distribution (heterogeneous or homogenous) and reproducibility of assessment, all cases were classified into three groups by paired-difference (d) between means of hot-spot score and average score (d<5, 5≤d<10, d≥10). The smaller the paired-difference between hot-spot score and average score, the more homogenous staining of Ki67 was indicated. ICC of three groups was evaluated. The consistency among five pathologists was observed to decrease with increasing paired-difference according to both methods (Table 4; Fig 4). Two score methods both showed a perfect correlation in d<5 (ICC, average score: 0.940 {95% CI 0.919, 0.957}; hot-spot score: 0.944 {95% CI 0.924, 0.960})and 5≤d<10 groups (ICC, average score: 0.835 {95% CI 0.760, 0.895}; hot-spot score: 0.816 {95% CI 0.735, 0.883}), and a substantial correlation in d≥10 group (ICC, average score: 0.748 {95% CI 0.616, 0.858}; hot-spot score: 0.602 {95% CI 0.437, 0.760}). A best correlation in d<5 group compared with 5≤d<10 and d≥10 groups was both demonstrated among five pathologists according to two score methods, indicating that pathologists may reach better Ki67 LI agreement in homogenous staining slides than in heterogeneous staining ones.

**Table 4:** The ICC and 95% CI on Ki67 LI, stratified by paired-difference between means of hot-spot score and average score.

	N	ICC (95% CI) for average score	ICC (95% CI) for hot-spot score
d<5	89	0.940 (0.919, 0.957)	0.944 (0.924, 0.960)
5≤d<10	44	0.835 (0.760, 0.895)	0.816 (0.735, 0.883)
d≥10	27	0.748 (0.616, 0.858)	0.602 (0.437, 0.760)

d: paired-difference between means of hot-spot score and average score

## Discussion

The nuclear protein Ki67 is often used as a marker of cellular proliferation, and is a good predictive and prognostic marker widely used in clinical practice of breast cancer [1–7]. Studies have demonstrated that Ki67 assessment helped to more reliably define prognosis in patients with ER-positive and HER2-negative breast cancers [2,4]. In patients received neoadjuvant chemotherapy, Ki67 LI is reported to be associated with pathological response [7]. The St. Gallen Consensus Meeting [8] determined that the Ki67 labeling index is important for distinguishing between ‘‘luminal A” and ‘‘luminal B (HER2-negative)” breast cancer subtypes, and advised adjuvant chemotherapy for luminal B but not for luminal A. Misinterpretation of the Ki67 labeling index may result in a lost opportunity for patients to receive chemotherapy or may result in patients being overtreated. Therefore, the standardization of the assessment of Ki67 LI is considered essential to critically evaluate the clinical value of Ki67 LI and to apply it in clinic.

There is no standard method yet to assess Ki67 in breast cancer, and there are still many technical issues to be resolved, such as how to count positive cells, how to select scoring areas. In 2011, the International Ki67 in Breast Cancer working group published recommendations for Ki67 assessment in breast cancer. The guideline aimed for better analysis, reporting, and use of Ki67 that should minimize interlaboratory variability and improve inter-study comparability of Ki67 results. This includes using an overall average score and scoring 1000 cancer cells, with 500 cells as the absolute minimum [14]. However, manual counting of as many as 1000 cancer cells is labor intensive and error prone. Counting many cells as part of a standard test could greatly slow its processing. Automated digital image analysis is a candidate [16], but not all institutes can afford it. Visual assessment of Ki67 LI is a simpler method, which would be easier and faster, is now used to evaluate Ki67 LI in a considerable number of pathological institutions and laboratories. Hida and his colleagues proposed a fast and easy method of visual assessment named “eye-10” to evaluate Ki67 [17]. Their study indicated that visual assessment of Ki67 at a glance was an easy method to exclude many luminal-type breast cancers from counting 1000 cells study, and to exclude obviously high and low Ki67 cases. In our study, we evaluated the interobserver concordance of visual assessment of Ki67 LI among five breast pathologists. All slides were visual estimated, and proportions of positive tumor cells at 10% intervals were scored. A perfect agreement of Ki67 LI was demonstrated among five pathologists. Our study indicated that visual assessment of Ki67 LI on IHC staining at 10% intervals is a candidate for a standard method; it is much faster and easier than manual counting, and simpler and less expensive than computer-based image analysis. This fast, cheap and easy method may have wide applications in breast cancer clinical practice.

Biological heterogeneity of Ki67 staining can occur across breast cancer specimens, and the location and extent of the area of the cancer that should be scored is controversial, which has been an important reason of the low interobserver reproducibility. Excellent concordance has been reported when the same area is counted using printed photographs [12]. The approach to scoring hot spots varies across studies. The International Breast Cancer working group recommended that hot spots be included in an overall average assessment of Ki67 across the whole section [14]. Our study evaluated the concordance with two different score methods among five pathologists. The pathologists used two different methods to choose the scoring fields: hot-spot assessment and average assessment including hot spots. A perfect concordance of Ki67 LI was both demonstrated among five pathologists according to two score methods. Average score method (ICC 0.904) showed a better concordance than hot-spot score method (ICC 0. 894). Our study indicated that an overall average assessment across the whole section including hot spots may be a better visual assessment method used in routine practice.

Our study also showed that the staining distribution of Ki67 was associated with the reproducibility of assessment. Ki67 staining was homogenous in some slides and heterogeneous in others. According to paired-difference between means of hot-spot score and average score of each case, all cases were divided into three groups (d<5, 5≤d<10, d≥10). Smaller paired-difference meant more homogenous in staining. The consistency among five pathologists was observed to decrease with increasing paired- difference according to both methods. A best agreement was demonstrated in d<5 group compared with 5≤d<10 and d≥10 groups among five pathologists. Our study indicated that biological heterogeneity of Ki67 staining is an important reason of the low interobserver reproducibility, and observers may reach better Ki67 LI agreement in homogenous staining slides than in heterogeneous staining ones.

Clinical decision-making in breast cancer treatment often relies on a Ki67 cutoff to classify patients into “Ki67 high” or “Ki67 low” risk groups. The choice of the cutoff has a major impact in practice, as it may determine which patients receive more aggressive therapy. However, discrete cutoff values to distinguish high from low Ki67 expression have not been clearly established and remain controversial [2,18–20]. Many cutoffs have been used, although 10%–30% have been the most common to dichotomize populations [2,18–20]. However, without standardization of methodology, these cutoffs have limited values in routine practice. Varga’s study suggested high interobserver variability in the intermediate Ki67 cases (Ki67 index of 8%-15%), precisely the range in which most cutoffs are located for making clinical decisions [19]. Hida’s study showed intermediate or “gray zone” categories (10%-20%) are generally less reproducible than low- and high-value categories by visual assessment [17]. In our study, a perfect correlation in low Ki-67 LI group (≤10%), a substantial correlation in high Ki-67 LI group (>30%), and a fair to moderate correlation in intermediate Ki-67 LI group (11%–30%) were observed among five pathologists by visual assessment. Concordance of intermediate Ki67 LI group (11%–30%) was relatively lower than low Ki67 LI group (≤10%) and high Ki67 LI group (>30%). Manual counting of 1000 cancer cells can be used in intermediate groups, but it is a tedious task for pathologists and has the question of reproducibility [10,19]. Other methods, such as automated digital image analysis, may also be used to define the intermediate groups [16]. In the absence of harmonized methodology, it might be better to keep the ‘‘intermediate” proliferation group as a “gray zone” and leaving decisions about adjuvant therapy up to other factors such as histological grade, nodal status, tumor size and patient preferences.

In conclusion, our study suggested that visual assessment of Ki67 LI on IHC staining at 10% intervals is a candidate for a standard method in breast cancer clinical practice. Average score and hot-spot score of visual assessment both demonstrated a perfect concordance of Ki67 LI, and an overall average assessment across the whole slide including hot spots may be a better visual assessment method in routine practice. Interobserver concordance of intermediate Ki67 LI in which most cutoffs are located for making clinical decisions was relatively low. Well-validated methodologies to evaluate the “gray zone” around the cutoff points of Ki67 may allow more accurate risk estimation and therefore better clinical management.

## Supporting Information

S1 Dataset The detailed data of scores of each pathologist. (XLS)
